# Mdm2-SNP309 polymorphism in prostate cancer: no evidence for association with increased risk or histopathological tumour characteristics

**DOI:** 10.1038/sj.bjc.6604441

**Published:** 2008-06-24

**Authors:** R Stoehr, F Hitzenbichler, B Kneitz, C G Hammerschmied, M Burger, A Tannapfel, A Hartmann

**Affiliations:** 1 Institute of Pathology, University Hospital Erlangen, Universitaetsstrasse 22, Erlangen 91054, Germany; 2 Institute of Pathology, University of Regensburg, Franz-Josef-Strauss-Allee 11, Regensburg 93053, Germany; 3 Department of Urology, University of Wuerzburg, Oberduerrbacherstrasse 6, Wuerzburg 97080, Germany; 4 Department of Urology, University of Regensburg, Franz-Josef-Strauss-Allee 11, Regensburg 93053, Germany; 5 Institute of Pathology, Ruhr-University of Bochum, Buerkle de la Camp –Platz 1, Bochum 44789, Germany

**Keywords:** prostate cancer, Mdm2 SNP309, RFLP, p53 pathway

## Abstract

The search for inherited cancer susceptibility factors is a major focus of epidemiologic cancer studies. Analyses of single-nucleotide polymorphisms (SNP) in a variety of genes revealed a correlation between a specific allele variant and cancer predisposition. Human mouse double-minute 2 protein (Mdm2) is a cellular E3 ligase capable of ubiquitination and degradation of p53. Therefore, Mdm2 is a crucial factor of cell cycle control and cell survival. The Mdm2 promoter SNP309 was shown to increase Mdm2 expression and can, thereby, inhibit the p53 pathway. This SNP was found to be associated with increased risk and early onset of various malignancies. For prostate cancer no studies are reported to date. In a case–control study we determined the distribution of the Mdm2 SNP309 in 145 male subjects with prostate cancer and in 124 male controls without any malignancy using RFLP analysis. Cases and controls showed a similar distribution of the SNP (*P*=0.299). Genotype distribution showed neither an association with histopathological characteristics of the tumours nor with prognosis. Age at disease onset was also not modified by the SNP. This first study of the Mdm2 SNP309 in prostate cancer patients suggests no correlation between a certain allelic variant and an increased cancer risk.

Genomic integrity and the ability of accurate repair of cellular damage are crucial prerequisites for controlled proliferation and differentiation. These processes are highly regulated and disruption of components of this network favours malignant transformation.

The p53 protein is one of the most important coordinators of cellular response to genotoxic stress (Levine 1997). The tumour suppression functions of p53 are widespread and mediated by various mechanisms. p53 is tightly regulated and the exact mechanisms of p53 activation are still not fully understood (Brooks and Gu, 2006). Several studies have shown that the major mechanism of p53 control is its degradation by the ubiquitin-proteasome pathway (Brooks and Gu, 2006). Human mouse double-minute 2 protein (Mdm2) (gene locus on chromosome 12q14.3-q15) is a cellular E3 ligase able to ubiquinate and degrade p53 (Haupt *et al*, 1997). As a key negative regulator of p53, overexpression of Mdm2 is associated with accelerated tumour progression and lack of response to therapy in various human malignancies (Bond *et al*, 2004). Besides gene amplification, a naturally occurring single-nucleotide polymorphism (SNP) in the promoter of the Mdm2 gene (SNP309, T>G) was found that increases mRNA and protein levels (Bond *et al*, 2004). Several studies have analysed the impact of the Mdm2 SNP309 on cancer risk in sporadic and hereditary malignancies and a recently published meta-analysis revealed that there is only little effect on the risk of common cancers from this SNP alone. Nevertheless, a modification of the onset of tumour formation and the individual prognosis might be influenced by this sequence variation (Wilkening *et al*, 2007). Interestingly, Bond and co-workers found an acceleration of tumour formation in a gender-specific and hormone-dependent manner by this polymorphism (Bond *et al*, 2006).

Prostate cancer (PCa) poses one of the major health care problems today. PCa is responsible for approximately 11% of all male cancers in Europe, and about 9% of all cancer deaths in men are caused by this malignancy within the European Union, the course of this malignancy is unpredictable and therapy not warranted in all patients (Heidenreich *et al*, 2008). A variety of treatment options ranging from watchful waiting to radical surgery underlines the necessity for the identification of reliable factors permitting individual risk assessment.

Major progress in discovery of SNPs has lead to a rapid increase of genetic association studies in various malignancies including PCa. Despite promising results no specific PCa-related genes have been established yet. However, the involvement of variants in androgen pathway genes in PCa aetiology could recently be confirmed reproducibly (Lindström *et al*, 2006).

Alterations in the p53 pathway are already described in PCa including Mdm2 protein overexpression in about 30–45% of the analysed tumours. This overexpression was associated with advanced tumour stage, higher tumour volume and increased cell proliferation (Osman *et al*, 1999; Leite *et al*, 2001; Khor *et al*, 2005). Although these data indicate a potential role of Mdm2 overexpression in PCa, there have been no studies published investigating the distribution of the functional Mdm2 SNP309 in PCa patients to date. Therefore, the aim of the presented study was the analysis of the allele frequencies of the Mdm2 SNP309 in patients with PCa compared to a healthy control group to assess a possible influence of this promoter alteration on cancer risk, histopathological tumour characteristics and prognosis.

## Materials and methods

### Samples

Overall, 145 patients with PCa were included in our study. All PCa patients underwent a radical prostatectomy. Formalin-fixed and paraffin-embedded tissue samples from these patients were available from prior transurethral resection (TUR-P, *n*=31) or from the prostatectomy specimens (*n*=114). For comparison 124 samples from a male control group of patients without any malignancy acquired at the Department of Urology, University of Regensburg was investigated.

All tumours were diagnosed according to the 2004 WHO classification of prostate tumours (Epstein *et al*, 2004) and staged according the TNM system (Sobin and Wittekind, 2002). Characteristics of the study participants are shown in Table 1. Detailed clinical information was available from 65 PCa patients. Clinical follow-up (mean 71 months and range 6–144 months) revealed that 20 tumours showed a recurrence within 3 years (defined as detectable serum prostate specific antigen (PSA) following radical prostatectomy (>0.2 ng ml^−1^), whereas 45 tumours did not recur within at least 5 years. Prior IRB approval was obtained for the study.

### Tissue microdissection and DNA isolation

DNA was extracted from normal prostate tissue or peripheral blood using the High Pure PCR Template Preparation Kit (Roche GmbH, Mannheim, Germany) according to the manufacturer's instructions.

### Mdm2 SNP309 analysis

Single-nucleotide polymorphism analysis was carried out by restriction fragment length polymorphism analysis (RFLP) of the promoter region which contains an *Msp* A1I site (5′-CMGCKG-3′) in presence of the G-allele (Sotomaa *et al*, 2005). The presence of the G-allele resulted in a digest of the PCR product (157/106+51 bp), PCR products containing the T-allele remained unaffected.

### Amplification of promoter region variants and RFLP analysis

Single-nucleotide polymorphism region was amplified by PCR using primers (5-FAM-sense: 5′-CGCGGGAGTTCAGGGTAAAG-3′; antisense: 5′-CTGAGTCAACCTGCCCACTG-3′) obtained from Metabion (Martinsried, Germany) in a total volume of 25 *μ*l containing approximately 100 ng DNA, 0.2 mM dNTP (Roche Diagnostics), 0.18 *μ*M primers and 0.0025 U *μ*l^−1^ GoTaq (Promega, Mannheim, Germany). The thermal cycling conditions were as follows: initial denaturation for 3 min at 95°C, 35 cycles of denaturation at 95°C for 1 min, annealing at 61°C for 1 min, elongation at 72°C for 1 min and final primer extension at 72°C for 10 min.

**Figure 1:**
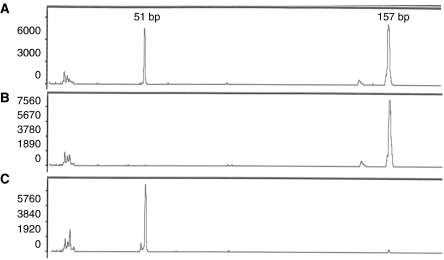
Representative examples for RFLP analyses. (**A**) T/G genotype resulting in an undigested 157 bp product and a digested 51 bp product. The second fragment from the *Msp* A1I digest (106 bp) is not visible due to 5′-FAM-labelling of the PCR product. (**B**) T/T genotype showing only the 157 bp PCR product after *Msp* A1I digest. (**C**) G/G genotype displays only the 51 bp after digest of the PCR product.

PCR products were incubated overnight with 5 U *Msp* A1I (New England Biolabs, Frankfurt/Main, Germany) and 100 *μ*g ml^−1^ BSA at 37°C in a total volume of 30 *μ*l to ensure complete digestion. Restriction fragments were separated by capillary electrophoresis using an ABI PRISM 310 genetic analyser (Applied Biosystems, Foster City, CA) and analysed with the GeneScan Analysis Software. Representative examples of genotyping are shown in Figure 1. Ten randomly selected cases were also sequenced to verify the RFLP results. In all cases identical results were obtained (data not shown).

### Statistical analysis

To test if the genotype distribution followed Hardy–Weinberg equilibrium, the public software at http://ihg.gsf.de/cgi-bin/hw/hwa1.pl was used. *χ*^2^ statistics (two-sided Fisher's exact test) were used to evaluate case-control differences in the distribution of genotypes and to analyse associations between genotypes and clinical or histopathological characteristics. *P*<0.05 was interpreted as statistically significant.

## Results

**Figure 2:**
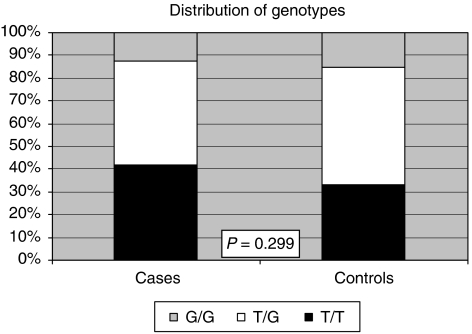
Graphical illustration of the genotype distribution in cases and controls.

All analysed samples gave interpretable results. The genotype distribution in our cohorts followed the Hardy–Weinberg equilibrium in cases (*P*=1.000) and controls (*P*=0.578). Genotype distribution between cases and controls did not differ significantly. Although there was a higher frequency of T/T genotypes in prostate cancer patients this difference did not reach statistical significance (*P*=0.299, Table 2, Figure 2). There was also no association between overall disease risk and presence of the polymorphic promoter variant (T/T *vs* T/G+G/G; *P*=0.131, Table 2). In addition, we also did not observe any significant correlation of genotypes and disease recurrence or Gleason score (Table 3). As the Mdm2 SNP309 was previously shown to be related with disease onset at younger age also in sporadic tumours (eg, bladder cancer (Sanchez-Carbayo *et al*, 2007)) we tested our cases towards this hypothesis. Within our cohort there was no significant association between genotypes and early disease onset (⩽60 years *vs* >60 years, Table 3).

## Discussion

The Mdm2 SNP309 is a plausible cancer predisposing allele due to the crucial role of Mdm2 in the cellular p53 pathway. The T to G variant was shown to result in increased Mdm2 synthesis and was found to be correlated with the risk of cancer or an early onset of tumour formation at various organ sites (Hu *et al*, 2007). In this study, we genotyped 145 cases of PCa and 124 male controls for this polymorphism. In our cohort, we did not find any association of the SNP309 with tumour risk, age at tumour onset, histopathological characteristics of the tumours or prognosis.

Overexpression of Mdm2 and its clinical consequences were already described in PCa (Osman *et al*, 1999; Leite *et al*, 2001; Khor *et al*, 2005) but the molecular basis of this upregulation is still unclear. As our study revealed no significant changes in the allelic distribution between PCa patients and men without any malignancy, it is unlikely that the Mdm2 SNP309 plays an important role for increased Mdm2 expression in prostate tumours. Moreover, chromosomal deletions at 12q have not been described in PCa to date, making a preferential loss of the Mdm2 T-allele resulting in a Mdm2 overexpression during PCa development unlikely (Dumur *et al*, 2003).

Mdm2 overexpression could be linked to gene amplification in several malignancies (eg, malignant melanoma, non-small cell lung cancer and lipomatous tumours (Dworakowska *et al*, 2004; Nilsson *et al*, 2004; Muthusamy *et al*, 2006) but in PCa a specific Mdm2 gene amplification could not be demonstrated by Southern blot analysis so far (Ittmann *et al*, 1994). Interestingly, frequent gains of chromosome 12q including the regions 12q13-q14 that are in close proximity to the Mdm2 gene locus have been reported in various studies using comparative genomic hybridisation (Sattler *et al*, 1999; Zitzelsberger *et al*, 2001) but to date no gene copy number analysis specific for Mdm2 using highly sensitive, fluorescence-based methods has been performed.

The G-variant of the Mdm2 promoter polymorphism increases the affinity of transcription factors for example, Sp1. As long as Mdm2 gene amplification is not analysed in detail in PCa, overexpression of Sp1 might be speculated as an activator of increased Mdm2 synthesis. Numerous studies have documented that Sp1 activity and/or Sp1 expression levels are elevated in various human cancers and are associated with prognosis (Safe and Abdelrahim, 2005; Deniaud *et al*, 2006). In PCa elevated Sp1 levels have not been described so far but an increased expression of Sp2 in PCa cells was found recently (Phan *et al*, 2004) indicating a possible role of deregulation of Sp/KLF family member expression in PCa.

Mdm2 is still discussed as a promising target for a therapeutical approach (Vassilev, 2007). The identification of the first selective and potent inhibitors of the p53-Mdm2 interaction emphasised the usage of small-molecule inhibitors as viable alternative to chemotherapy for selective p53 activation in tumours with wild-type p53. In PCa p53 alterations are a rare event in primary tumours and associated with more aggressive disease, metastasis and transition from androgen-dependent to androgen-independent growth (Dong, 2006). Therefore, a subset of prostate tumours with Mdm2 overexpression might evolve as a suitable target for the application of selective Mdm2 inhibitors in the future.

In conclusion, our case–control study suggests that the Mdm2 promoter polymorphism SNP309 has no influence on PCa risk or prognosis, and relevant inherited alterations in PCa-related genes remain to be uncovered.

## Figures and Tables

**Table 1:** Characteristics of study participants

	**Cases**		**Controls**	
Number:	*n*=145		*n*=124	
*Age*:	Median: 65	Range: 46–74	Median: 69	Range: 40–88
	Mean: 64.2 (±6.0)		Mean: 68.5 (±9.9)	
*Stage*:	pT1-3a	*n*=105		
	pT3b-3c	*n*=28		
	No data available	*n*=12		
*Gleason score:*	Median: 7	Range: 3–10		
*Gleason sum:*	3–4	*n*=3		
	5–7	*n*=103		
	8–10	*n*=27		
	No data available	*n*=12		
*Recurrence*:	Recurrence within 3 years	*n*=20		
	No recurrence within 5 years	*n*=45		
	No data available	*n*=80		

**Table 2:** Distribution of allelic variants between participants

**Genotype**	**Cases (%)**	**Controls (%)**	**Comparison**		
TT	61 (42.1)	41 (33.1)			
GT	66 (45.5)	64 (51.6)	*P*=0.299		
GG	18 (12.4)	19 (15.3)			
TT	61 (42.1)	41 (33.1)	*P*=0.131	OR: 0.680	95% CI: 0.413–1.120
TG+GG	84 (57.9)	83 (66.9)			

**Table 3:** Distribution of allelic variants between relevant tumour/patient characteristics

**Genotype**	**Cases with recurrence (%)**	**Cases without recurrence (%)**	**Comparison**		
TT	8 (40)	13 (28.9)			
GT	10 (50)	27 (60)	*P*=0.688		
GG	2 (10)	5 (11.1)			
TT	8 (40)	13 (28.9)	*P*=0.402	OR: 1.641	95% CI: 0.545–4.943
TG+GG	12 (60)	32 (71.1)			

	**Gleason sum <7 (%)**	**Gleason sum ⩾7 (%)**			
TT	19 (35.9)	34 (43)			
GT	27 (50.9)	36 (45.6)	*P*=0.726		
GG	7 (13.2)	9 (11.4)			
TT	19 (36)	34 (43)	*P*=0.471	OR: 0.740	95% CI: 0.361–1.514
TG+GG	34 (64)	45 (57)			

	**Age ⩽60 (%)**	**Age >60 (%)**			
TT	11 (35.5)	40 (42.1)			
GT	15 (48.4)	46 (48.5)	*P*=0.533		
GG	5 (16.1)	9 (9.5)			
TT	11 (35.5)	40 (42.1)	*P*=0.537	OR: 0.756	95% CI: 0.326–1.753
TG+GG	20 (64.5)	55 (57.9)			

CI=confidence interval; OR=odds ratio.
